# Quantifying the scientific revolution

**DOI:** 10.1017/ehs.2023.6

**Published:** 2023-04-13

**Authors:** Benoît de Courson, Valentin Thouzeau, Nicolas Baumard

**Affiliations:** 1 Max Planck Institute for the Study of Crime, Security and Law, Günterstalstraße 73, 79100 Freiburg, Germany; 2 Ecole Normale Superieure, Departement d'Etudes Cognitives, Departement d'Etudes Cognitives, Paris, France

**Keywords:** Cultural evolution, history of science, digital humanities, economic development

## Abstract

The Scientific Revolution represents a turning point in the history of humanity. Yet it remains ill-understood, partly because of a lack of quantification. Here, we leverage large datasets of individual biographies (*N* = 22,943) and present the first estimates of scientific production during the late medieval and early modern period (1300–1850). Our data reveal striking differences across countries, with England and the United Provinces being much more creative than other countries, suggesting that economic development has been key in generating the Scientific Revolution. In line with recent results in behavioural sciences, we show that scientific creativity and economic development are associated with other kinds of creative activities in philosophy, literature, music and the arts, as well as with inclusive institutions and ascetic religiosity, suggesting a common underlying mindset associated with long-term orientation and exploration. Finally, we investigate the interplay between economic development and cultural transmission (the so-called ‘Republic of Letters’) using partially observed Markov models imported from population biology. Surprisingly, the role of horizontal transmission (from one country to another) seems to have been marginal. Beyond the case of science, our results suggest that economic development is an important factor in the evolution of aspects of human culture.

## Graphical abstract

**Figure:**
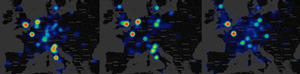

The Scientific Revolution represents a turning point in the history of humanity. In the space of a few decades, it transformed the nature of knowledge and the capacities of humankind (Cohen, 2012; Mokyr, 2016; Wootton, 2015). ‘Without it’, writes historian of science David Wootton, ‘there would have been no Industrial Revolution and none of the modern technologies on which we depend; human life would be drastically poorer and shorter and most of us would live lives of unremitting toil’ (Wootton, 2015).

A large number of explanations have been put forward to explain the origins of the Scientific Revolution (for a review, see Cohen, 1994), from the belief in a divine legislator (Grant & Grant, 1996; Needham, 1981; Stark, 2007) to the role of medieval universities (Grant & Grant, 1996; Huff, 2017) and from political freedom (Mokyr, 2016; Needham, 1981) to the invention of the printing press (Eisenstein, 1980; Wootton, 2015) So far, ‘there is no general agreement on what the Scientific Revolution is, why it happened – or even whether there was such a thing’ (Wootton, 2015).

This lack of consensus comes arguably at least in part from the absence of quantitative data: it is hard to rule in or out a candidate explanation with purely qualitative data. However, the growing size of online datasets makes it henceforth possible to estimate cultural production over time in science as well as in the arts (Fraiberger et al., 2018; Gergaud et al., 2017; Schich et al., 2014; Serafinelli & Tabellini, 2022); Sinatra et al., 2016). In particular, Wikipedia has become the largest database of biographies, and offers reliable and well-edited data on people's demographic features (gender, lifespan, nationalities), occupations and cultural production (Baumard et al., 2018; Gergaud et al., 2017).

In this dtudy, we gathered the Wikipedia pages of all individuals classified as scientists during the early modern period: mathematicians, astronomers, physicists, biologists, naturalists, chemists, botanists, entomologists and zoologists (see Table S1). Then, we estimated the scientific contribution of each of these 6620 individuals through different proxies (the size of the page, the number of translations in other languages and the number of Wikipedia pages containing a link to this page). With such a large dataset, we can go beyond key figures such as Newton and Galileo, and take into account the thousands of little-known individuals who contributed to the rise of science (see Table S1) Put otherwise, it allows us to incorporate not only the discovery of the law of gravitation or the moons of Jupiter, but also the hundreds of mathematical theorems and astronomical discoveries that paved the way for the major breakthroughs of the Scientific Revolution. All these information are associated with academic references (Mesgari et al., 2015; Nielsen, 2007; Willinsky, 2010).

In the burgeoning field of scientometrics, two foundational papers (King, 2004; May, 1997) have shown that the scientific production of modern countries over a period of time can be estimated by the quantity and the impact of the papers published by the scientific community of a country. Just as gross domestic product (GDP) is a measure of economic wealth, the average impact of a scientific paper could be used as a measure of the scientific wealth of nations (Adams, 1998; Albarrán et al., 2010; Holmgren & Schnitzer, 2004; King, 2004; May, 1997).

The aim of this paper is to build on these works, and extend their approach to the pre-modern period in order to better understand the origins of the Scientific Revolution. Counting publications and citations is obviously complicated for ancient periods. However, data sources like Wikipedia also carry an inherent estimate of a scientist's importance: the larger an individual's output, or the more important it is in the eyes of moderns, the higher we expect the Wikipedia indicators (page length, number of links pointing to that page, number of languages with a version of that page) to be (see Table S1). As such, these estimates are very similar to the modern scientometric evaluations of scientific productivity that are based on citations index (i.e. Science Citation Index, PubMed, Web of Science, Scopus, GoogleScholar; Aksnes et al., 2019; Alonso et al., 2009; Archambault et al., 2009; Falagas et al., 2008; Harzing & Alakangas, 2016; Mongeon & Paul-Hus, 2016). Indeed, the more important a scientist's contribution is, the more pages of later generations of scientists in Wikipedia will link to this page (for example, many pages of evolutionary biologists link to Darwin's page). Thus, we used Wikipedia's estimate of the importance of scientific contributions to create aggregate estimation of scientific production of countries over time (see Figures S1 and S2). Note that, throughout the article, we use modern borders to define geographical areas of interest. This is a standard method in economic history for estimates of population size, GDP and urbanisation (see for instance (Bosker et al., 2013; Broadberry et al., 2015; McEvedy et al., 1978).

Estimates based on Wikipedia are the product of thousands of editors, using tens of thousands of academic references. Thus, they provide the most neutral estimate of the current consensus in the history of science about the importance of a specific scientific contribution. For instance, the page for Galileo has been edited by 2931 different editors (excluding bots) since its creation in 2001. Even the pages of minor figures such as Jean de Hautefeuille (1646–1724, French physicist), Thomas Johnson (1595–1644, English botanist) or Joachim Jungius (1597–1657, German mathematician) have been edited by more than 30 different contributors.

Another advantage of Wikipedia is that the database has not been built with a particular hypothesis in mind. For instance, for each individual, Wikipedia provides one or several ‘occupations’ (writer, painter, theologian, etc.). This label allows us to distinguish between individuals who truly contributed to science (e.g. Newton) and those who merely commented on or recorded the advances of science (e.g. Roger Bacon, Thomas Huxley) without biasing the sample. Importantly, these choices were not influenced by the goal of this study.

Obviously, just as modern estimates (Aksnes et al., 2019; Alonso et al., 2009), an estimate based on Wikipedia has its advantages and its drawbacks (see below). For instance, in Wikipedia, some very notable people such as Goethe are included in lists of scientists because of their scientific contributions (although these are marginal in regard to their non-scientific contributions). It could thus be the case that the results were biased by these non-scientific celebrities. In an additional analysis, we only included individuals whose primary occupation as listed on their Wikidata item page was as a scientist (in one of the categories specified above). This did not substantially change the pattern (see Figure S7).

Another bias of Wikipedia is that the scientific contributions of the English are probably overestimated owing to the size of the English-speaking community both in American history of science and among Wikipedia contributors (Callahan & Herring, 2011; Gergaud et al., 2017). Given the current size and influence of American and other English-language communities of historians of science compared with French-, German- or Italian-speaking communities, it could be the case that scientific contributions made by English people have been overestimated, because they are more easily accessible to English-speaking historians than the scientific contributions made in other languages. To counteract this potential bias, we used the three Wikipedias (English, German and French) whose Wikipedia communities had established lists of writers, scientists, and artists by century and nationality (in line with Gergaud et al., 2017). Combining Wikipedia entries from several different languages allows us to limit anglocentric bias. We also carried out a control analysis without the English Wikipedia. Including or excluding the English Wikipedia does not change the results (see Figure S10).

Although national contribution to world science is clearly an important indicator, it is crucial to compare scientific outputs relative to population (King, 2004; May, 1997). Today, for instance, the US, China, Japan and the UK are prominent contributors to world science. Yet the size of their population hides the fact that some countries such as the Scandinavian countries, Israel and Switzerland are in fact more productive than the great scientific nations (King, 2004; May, 1997). In the same way, it is usual to consider that, in the modern period, Italy (with Galileo and Torricelli), England (with Newton and Boyle) and France (with Pascal and Descartes) were more or less equally productive scientifically (see Figure S2). Yet this assessment overlooks the fact that in 1650, France had 16 million people, Italy 12 million and England only 3 million. In other words, for England to contribute as much to the Scientific Revolution, it had to be much more productive than France or Italy. Current qualitative estimates of total national productivity fail to capture the differential of productivity between Italy, England and France.

In this paper, we explore the role of economic development, an important factor in cultural evolution (Baumard, 2017; Inglehart, 2018; Welzel, 2014) that has been relatively neglected in the history of the Scientific Revolution (Cohen, 1994). One of the reasons for the relative neglect is that it has long been thought that economic development was stagnant before the Industrial Revolution (Clark, 2008). However, recent works in economic history have demonstrated that north-western Europeans, and English people in particular, enjoyed an unprecedented level of living standard from the seventeenth century onward (Fouquet & Broadberry, 2015). English people were richer, healthier, taller, better nourished and better equipped than individuals in any previous society (Allen et al., 2011; Cummins, 2014; Fouquet & Broadberry, 2015; Morris, 2013).

Economic development might be crucial for the cultural evolution of science. First, quantitative works on modern countries have demonstrated that GDP per capita is strongly correlated with scientific productivity (Allik et al., 2013; Grossetti et al., 2014; King, 2004; Meo et al., 2013; Vinkler, 2008). Second, recent advances in behavioural and social sciences have shown that affluence has predictable effects on human psychology, risk-taking, exploration and creativity (for a recent review, see (Haushofer & Fehr, 2014; Pepper & Nettle, 2017), and can alter the dynamics of cultural evolution (André & Baumard, 2020; Baumard et al., 2015; Inglehart, 2018). Basically, when humans have met their basic needs for survival, they can turn to more long-term-oriented goals such as learning and exploration (Maslow, 1943). In line with this idea, research inspired by life-history theory has shown that in a harsh environment, when the levels of available resources are low and unpredictable, individuals tend to be more short-term-oriented, less exploratory and more conservative because exploration is both risky and costly. In contrast, in a resource-rich environment, individuals are future-oriented, more exploratory and more open-minded because they have more resources to cope with the inherent costs of exploration and learning (Haushofer & Fehr, 2014; Jacquet et al., 2019; Pepper & Nettle, 2017). Since science relies heavily on exploration, this predicts that a higher level of resources should be associated with a higher level of scientific creativity (Baumard, 2018). This prediction contrasts with a common assumption in cultural evolution according to which population size is crucial in explaining creativity (Collard et al., 2013; Henrich, 2004; Kremer, 1993; Shennan, 2001).

Interestingly, this behavioural framework makes further predictions (Baumard, 2018). First, scientific creativity should be associated with artistic creativity, because both science and the arts rely on exploration. Second, inclusive (Robinson & Acemoglu, 2012), more democratic institutions are also likely to be associated with scientific creativity because trust is a long-term investment and democratic institutions require a high level of trust. In fact, a growing body of works both in political science (Abramson & Boix, 2014; Boix & Stokes, 2003) and in the behavioural sciences (Hörl et al., (2020); Petersen & Aarøe, 2015; Safra et al., 2017) suggests that better life conditions are associated with higher level of interpersonal trust and higher support for inclusive institutions.

## Results

### The evolution of scientific production

**Figure 1.**
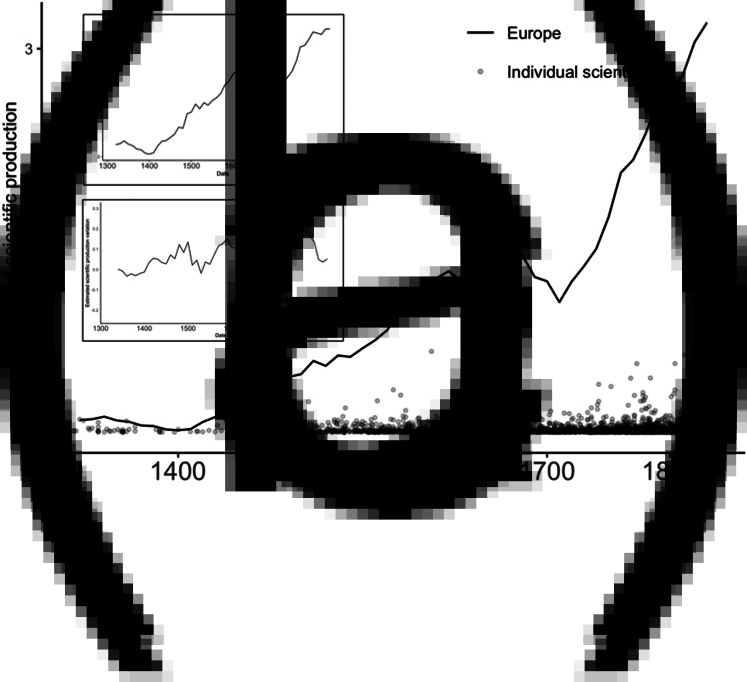
Evolution of scientific production (1300–1850). A) Total scientific production. Black points represent individual scientists' estimated production. The black line represents the log-transformed aggregated scientific production, computed as the sum of the individual scientists estimated production., B) Scientific production per capita, C) Growth rate in scientific per capita.

Our estimates show an increase of scientific production in Europe during the period 1300–1850, except for a period of crisis from 1690 to 1730 (see Figure 1a). Interestingly, the increase in scientific production does not seem to be higher during the seventeenth century than during the period before and after the Scientific Revolution. Our estimates also show an increase in scientific production per capita, suggesting that the European population becomes more productive over time (see Figure 1b). In both cases, the growth rate in production and production per capita is regular over the period, except for a period of crisis at the end of the seventeenth century (see Figure 1c for growth rate in per capita production). Overall, this suggests that the scientific revolution corresponds less to an acceleration of science than to an accumulation of discoveries until a point during the seventeenth century when scholars began to realise that they had reached a level of knowledge unprecedented in human history (Wootton, 2015).

**Figure 2.**
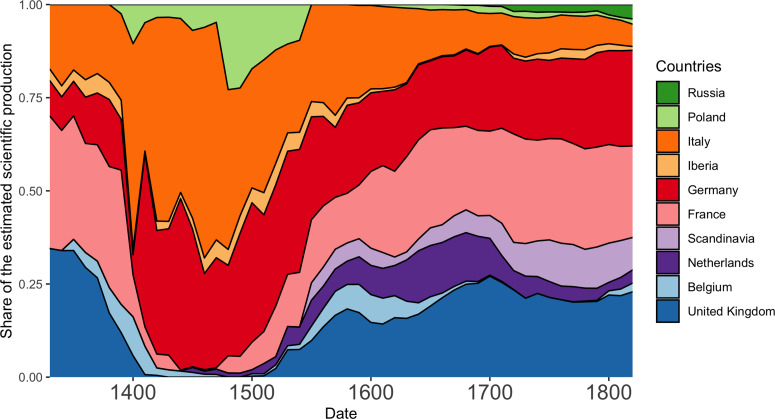
Evolution of the share of estimated scientific production per country (1300–1850). In Figure S10, we divide this plot between mathematics, physics and biology.

We now partition the scientific production into countries. Just like for modern science (King, 2004; May, 1997), this analysis reveals striking differences between countries, with four countries (Italy, Germany, the UK and France) accounting for over 80% of the European production (see Figure 2). Although these countries dominate the scientific production in all disciplines, there are slight differences between countries. For instance, compared with other countries, France has a larger share in mathematics than in physics and biology (see Figure 2b–d). Since France still has a larger share in mathematics today (King, 2004; May, 1997), this reveals the existence of very long national scientific traditions.

### Per capita national scientific production

**Figure 3.**
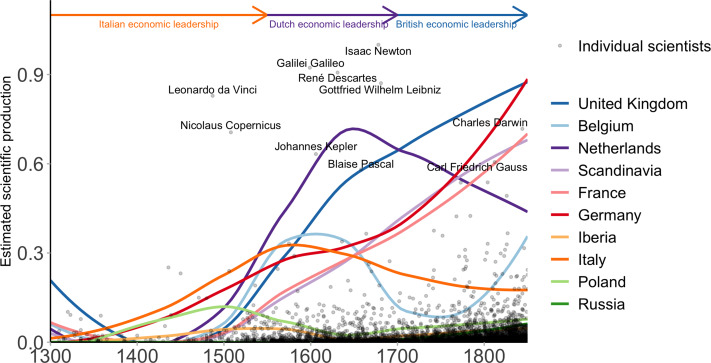
Scientific Production per capita (1500–1850). Black points represent individual scientists' estimated production, with the 10 highest scoring individuals labelled. The coloured curves represent the log-transformed aggregated scientific production per country, computed as the sum of the individual scientists estimated production over a window of 50 years, divided by their population at the time and smoothed by a local regression algorithm (loess). The economic leadership of Italy (orange), the Netherlands (purple) and the United Kingdom (blue) in terms of per capita GDP (>1500 dollars per capita and positive growth) coincides with their leadership in per capita scientific production.

We calculated estimates of per capita National Scientific Production in different countries (see Figure 3) with the Netherlands leading in the seventeenth century, followed by England in the eighteenth century. During the Scientific Revolution, these countries were much more productive than Germany, France or Italy on a per capita basis. It is worth noting that the high productivity of the Netherlands is visible both across indices (bytes, languages, quotations) and across Wikipedia languages (see Figure S3). During their Golden Age, the Dutch were more productive than the rest of Europe regardless of the choice of indicator. This is also the case for England in the eighteenth century, with the exception of two of the German indicators.

**Figure 4.**
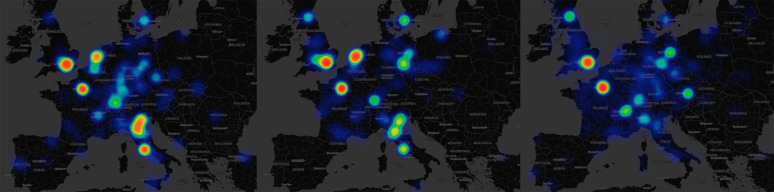
Geographic distribution of scientific production (left: 16th c., center: 17th c., right: 18th c.).

These results are in keeping with more qualitative modern assessments by historians of science (Rossi, 2001; Wootton, 2015). The relative decline of Italy is also visible from the early seventeenth century on, starting during Galileo's lifetime. The late rise of Germany's scientific productivity is also visible, and foreshadows the success of German science in physics, technology and chemistry at the end of the ninteenth century. Finally, although Scandinavia remained a small player during the early modern period owing to its small population size, it was among the most productive countries from 1700 onward. The same trends are also visible in the spatial distribution of scientists. As Figure 4 shows, scientific production was extremely concentrated in the most dynamic cities (Rome, London, Paris, Amsterdam, Florence and Venice) and shifted towards the north-west during the seventeenth and eighteenth centuries.

The picture painted by these results differs considerably from the one derived from national totals (see Figure S2). National estimates are distorted by the population size of countries. If we were to use them in 2021, they would highlight the production of China rather than Switzerland or Singapore. Another important take-home message from these results is that scientific productivity is highly variable from one period to the other. For instance, the Netherlands were much less productive in the sixteenth and eighteenth century than during their Golden Age (seventeenth century). Similar observations could be made about all the main players of the Scientific Revolution. This suggests that there are no permanent ‘national traits’, no countries that are intrinsically more scientific than others, but rather transient factors of scientific productivity.

We hypothesised that the results might be biased either by the top scientists (Newton, Huygens, etc.) or by the bottom scientists (the unknown 90%). Since the English Wikipedia is bigger than the French and German Wikipedia, this might lead to more English top scientists (because they are more visible, and have more translations) or more English bottom scientists (because there are more editors, which translates into a higher number of pages). To find out whether this was the case, we performed the same analysis for the top 10% of scientists and for the bottom 90%. We found that the pattern remained very similar in both cases (see Figures S4 and S5), in line with similar studies on contemporary countries (King, 2004).

Another potential bias is due to the fact that England and the Netherlands are smaller than Italy, France and Germany. It could thus be the case that their high productivity was due to the fact that similarly highly productive areas in France (such as Ile-de-France) or Italy (such as Tuscany) were drowned within bigger areas. To account for this possibility, we combined England, Scotland, Wales, Ireland, the Netherlands and Belgium into a larger unit as ‘north-western Europe’, with a combined population of 12.5 million people in 1700 (compared with 22 million for France, 13 million for Germany and 13 million for Italy). The pattern, although less spectacular, is substantially similar: north-western Europe comes out as the scientific leader from 1500 to 1800 (see Figure S6).

Another potential bias is that, in Wikipedia, some very notable people such as Goethe are included in lists of scientists because of their scientific contributions (although these are marginal in regard to their non-scientific contributions). It could thus be the case that the results were biased by these non-scientific celebrities. In an additional analysis, we only included individuals whose primary occupation as listed on their Wikidata item page was as a scientist (in one of the categories specified above). This did not substantially change the pattern (see Figure S7).

### Scientific productivity and economic development

Our results suggest that economic development and living standards are key to explaining scientific productivity, in line with similar studies on contemporary countries (Allik et al., 2013; Grossetti et al., 2014; King, 2004; Meo et al., 2013; Vinkler, 2008). To further investigate this relationship, we tested the association between scientific production and two proxies for living standards: GDP per capita and urbanisation rate. To investigate the link between environmental variables and scientific production, we built autoregressive linear mixed models using the R package spaMM (Rousset, 2017), whose results are displayed in Table 1. The dependent variable is the scientific production per capita, measured for each country every 50 years (to avoid any overlap between the datapoints). The scientific production values being overdispersed, we applied a logarithmic transformation. Each model has two fixed effects: first are the ones mentioned in the columns, whose coefficient and associated *p*-value figure in Table 1 (population, GDP per capita, urbanisation, number of universities per capita); second, we added the date as a covariate to (1) control for a potential historiographic bias, overstating the scientific production in the latest studied period because of the abundance of sources, and (2) to avoid a spurious correlation between affluence and scientific production, owing to the fact that both affluence and scientific production tend to increase with time. We added the country as an autoregressive random effect, to account for the fact two data points coming from the same country are not independent, in particular when they represent two consecutive time steps.

**Table 1.** Associations between environmental variables and cultural productions. Each cell represents a different model, with the row indicating the dependent variable and the column the independent one

	Population	GDP per capita	Urbanisation rate	Universities per capita	Parliament	Protestant
Scientists	−0.02	0.31**	0.16	0.05	0.42***	0.47***
Composers	−0.03	0.22	−0.11	−0.09	0.12	0.33*
Painters	−0.04	0.46***	0.68***	0.33*	0.13	0
Philosophers	0.06	0.17	0.29 0.	0.24	0.14	0.08
Writers	0.05	0.19	0.23	0.12	0.23	0.19
Sculptors	0.16	0.45**	0.42*	0.24	−0.04	−0.14
All	−0.03	0.44***	0.48***	0.23 0.	0.31*	0.17

We report the regression coefficient, and the *p*-value of a *t*-test:* *p* < 0.05,** *p* < 0.01,*** *p* < 0.001.

The results show a strong association between per capita scientific production, per capita GDP and urbanisation. Remarkably, the period of Italian, Dutch and English domination matches their period of maximum affluence during the studied period (i.e. the period during which their GDP per capita was over $1500 and their economic activity was growing, see Figure 3). Our dataset also allows for exploring alternative hypotheses, although better data would be required to test these hypotheses properly. We first considered the role of printing. Printing has often been cited as a cause of the Scientific Revolution, as it allows for better communication between scientists (Eisenstein, 1980; Febvre & Martin, 1997; Wootton, 2015). Although the increase in scientific productivity coincides roughly with the invention and diffusion of printing in Europe, it is notable that the invention of the printing press does not lead to the acceleration of scientific production. This result is in line with what can be observed outside Europe. For instance, the diffusion of printed books did not drastically change China's scientific productivity during the Tang dynasty (Xu, 2017), or that of the Ottoman Empire in the seventeenth century (Coşgel et al., 2012). In contrast, it is worth noting that Ancient Greece achieved a level of productivity often deemed similar to that of early modern Europe despite the absence of printing (Leroi, 2014; Russo, 2013).

We also examined the impact of universities. Medieval universities are often seen as an institutional innovation, acting as a driver of scientific improvement (Grant & Grant, 1996; Huff, 2017). This hypothesis is particularly favoured when comparing European institutions of higher education and scholarship with their equivalents in Muslim, Indian and Chinese societies (Grant & Grant, 1996; Huff, 2017). Moreover, it is true that the timing of the take-off of European science matches the creation of the major European universities (Bologna, Paris, Oxford). To test this hypothesis, we extended our study to the medieval period (500–1500). We found no significant association between scientific productivity and number of universities, suggesting that universities did not in fact play an important role in the Scientific Revolution (see Table 1).

One important hypothesis in cultural evolution is that the probability of innovations being discovered and maintained through social learning is greater in large populations than in small ones (Collard et al., 2013; Henrich, 2004; Kremer, 1993; Shennan, 2001; Vaesen et al., 2016). Our results, however, suggest that, at least in science, population size does not play an important role in explaining per capita scientific productivity. Small societies such as England, Holland and Scotland, despite their limited population, were much more innovative than larger societies such as France, Italy and Russia (see Table 1). This suggests that creativity is more important in determining the pace of cultural evolution than has been previously thought (André & Baumard 2020; Fogarty et al., 2017).

### A common creativity factor

**Figure 5.**
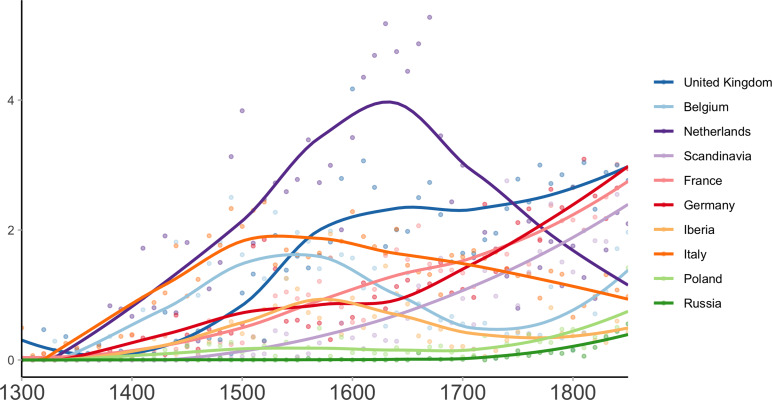
Estimated cultural production per capita (1500–1850).

Recent work in behavioural sciences has shown that innovation and creativity are strongly associated with affluence, whereas harsh and unpredictable environments lead to informational conformism, the tendency to defer to others’ judgments (Baumard, 2018; Inglehart & Welzel, 2005; Jacquet et al., 2018; Nettle, 2018). This behavioural approach to creativity suggests that individuals in affluent environments should be more innovative in science, but also in all kinds of activities, because the trade-offs between exploration and social learning are similar (Baumard, 2018). We therefore look at other kind of creative activities, such as literature, philosophy, painting, sculpture and music, and build a general per capita ‘Cultural Domestic Product’. In this index, scientists account for a small minority of the sample (19.9%), compared with painters (32.9%), writers (29.8%) and musicians (14.4%) (see Table S1). The pattern for cultural production is strikingly similar to the pattern of scientific production, with the Netherlands and then Britain leading (see Figure 5). This suggests a common general cause – a ‘culture of growth’ (Mokyr, 2016) – behind this high level of scientific, literary, and artistic production.

We also look at the correlation between artistic production and living standards using an analogous method. The results reveal a strong association between per capita cultural production and per capita GDP, urbanisation (see Table 1). Finally, to test the prediction that all forms of cultural productions are associated with a common underlying creativity factor, we used the same autoregressive linear mixed models to measure how much the cultural productions predict one another, while controlling for time and country (see Table 2).

**Table 2.** Associations between the different cultural productions

	Scientists	Composers	Painters	Philosophers	Sculptors	Writers
Scientists		0.12	0.39***	0.59***	0.28*	0.57***
Composers	0.1		−0.07	0.01	−0.01	0.1
Painters	0.46***	−0.07		0.71***	0.68***	0.56***
Philosophers	0.25**	−0.11	0.36***		0.22*	0.48***
Sculptors	0.11	−0.02	0.35***	0.25*		0.22**
Writers	0.58***	0.11	0.51***	0.83***	0.4**	

To test the association with inclusive institutions, we used the the number of calendar years per century in which for the various areas a parliament (or estates-general, cortes, corts, diet, sejm, riksdag, Generallandtag or Reichstag) assembled for official sessions during shorter or longer periods in a year (Van Zanden et al., 2012). In line with the life-history framework, our results show a strong association between per capita scientific production and the activity of parliaments.

### Modelling the role of economic development in the cultural evolution of science

We further investigate the importance of economic development by systematically studying the role of economic development and cultural transmission in a series of models. At first sight, cultural transmission should be key in explaining the variation in scientific productivity. Science is a cumulative process (Mesoudi, 2011; Mesoudi & Thornton, 2018). As Bernard of Chartres (and Newton after him) famously said, scientists ‘stand on the shoulders of giants’. Yet the history of science shows that the cultural transmission of science from one period to the other, or from one region to the other, is not automatic. The Europeans inherited the Greek and the Arab science, but with few exceptions, they did not really start being scientifically productive before the sixteenth century. In the same way, while Spain and Portugal were culturally close to France and Italy, they played a very limited role during the Scientific Revolution. These observations suggests that while the the ‘Republic of Letters’ was an important proximate cause, it might not be the ultimate factor in explaining the rise of science in early modern Europe.

Modelling the process of cultural transmission is particularly complex. For example, a region that produces a lot of scientific knowledge at a given time may lead a neighbouring region to produce a little more. This second region may in turn influence the scientific production of the first region by feedback. Also, economic development is likely to influence both the level of production, and the level of learning because higher economic development is associated with more investment in human capital and communication technology.

It therefore seems difficult to reconstruct the evolution of scientific production with simple statistical models. However, there are statistical inference methods, developed primarily in population biology, which allow us to study non-linear phenomena at the population level, including random processes and interactions between spatially distributed populations over time (see for instance Stocks et al., 2020). This method is particularly well suited to the study of the evolution of scientific productivity, since the productivity of a region of the world is likely to depend both on its own past productivity (e.g. through the knowledge stored in books) as well as on the output of neighbouring regions (e.g. through the diffusion of books from one country to its neighbours).

In terms of statistical properties, the data points we study are neither temporally independent (a country's scientific production at some time might depend on his own earlier production), nor spatially independent (the Netherlands’ scientific production might have influenced Belgium's, for instance). This means that to account for our data, a model should allow for (1) autocorrelation, (2) vertical diffusion (from one period to the next, within the same country) and (3) horizontal diffusion (influence of two neighbouring countries on each other). Besides, the fat-tailed nature of the data makes gaussian statistics unsuitable.

Several non-exclusive hypotheses can be proposed to explain its dynamics: a productivity based on random phenomenon (1), a productivity based on the recent production of science in the region (2), a productivity influenced by economic development (3), a productivity influenced by the past production of science in the region (4), a productivity influenced by the recent production of science in neighbouring regions (5), a productivity influenced by the past production of science in neighbouring regions (6), and an influence of other regions mediated by economic development (7). We constructed 11 models with different combinations of hypotheses 1–7, to represent the dynamics of cultural productivity over time (see Figure S11), in order to determine the most credible hypotheses to explain the data using Markov chain methods (King et al., 2016). The method and the results are described more thoroughly in the Supplementary Materials. A very large number of models can be proposed, and we have chosen to evaluate only a subset of the plausible combinations of processes responsible for scientific production, focusing on the potential role of the GDP on scientific production.

To evaluate best models the history of science, we use two indicators: model comparison using the AIC (Akaike Information Criterion) and BIC (Bayesian Information Criterion) (Table 3), and, when applicable (i.e. for nested models), hypothesis testing by a likelihood ratio test (Table S4). All methods yield the same picture. In Table 3, we also report the scaled estimated effect of the covariates for all the models.

**Table 3.** Scaled coefficients for covariates of the Pomp models, and information criterions for these models

	GDP per capita	Diff	GDP per capita × Diff	Cum	GDP per capita × Cum	Cum_diff	AIC	BIC
A							304.8	321.4
B							148.9	168.3
C	0.07						136.9	159.1
D				0.09			150	172.1
E	0.074				0.004		138.9	163.8
F		0.025					148.7	170.9
G	0.065	0.009					138.6	163.5
H			0.042				148.4	170.6
I	0.068		0.013				138.5	163.4
J						0.029	145.9	165.3
K	0.086	−0.087	−0.067	−0.237	−0.1	0.184	138.8	172

‘Diff’ stands for horizontal diffusion (the sum of productions of neighbouring countries divided by the squares distance between capital cities). ‘Cum’ stands for the cumulative estimated scientific production of a country before that time – i.e. vertical diffusion. ‘Cum_diff’ stands for the cumulative horizontal diffusion. The models A and B are excluded, as they use no covariate. The coefficients of model K are difficult to interpret, some covariates like horizontal diffusion having a negative effect. This is due to high covariance between covariates, especially when interactions are included.

In these models, economic development is the only variable which helps to explain the scientific production data. Our results reject the idea that horizontal diffusion is an important parameter in explaining scientific productivity during the early modern period: if added alone, its impact is estimated to be much lower than GDP per capita (0.025 vs. 0.07), and when added alongside GDP per capita, its effect disappears (Table 2). In both cases, the addition of horizontal diffusion to the model increases AIC and BIC (Table 2), and fails to reject the simpler model in log-likelihood tests (D vs. B, *p* = 0.50; E vs. C, *p* = 0.86). Similarly, the cumulative production over the entire past (the variable Cum) does not helps to account for scientific production (Italy after the seventeenth century is a case in point). Knowing only the productivity in the immediately preceding time interval and the GDP per capita seems to be the best way to predict scientific production in our sample.

From these results, one should not conclude that the transmission of information was not important during the Scientific Revolution and that the exchange of ideas was useless, but rather that doing science probably requires a certain level of economic development. This would explain why, despite their proximity to Germany and the influence of the German culture, Poland and Hungary did not develop vibrant scientific communities until the ninteenth century and their economic take-off (Bukowski et al., 2019). In the same way, this would explain why, after a flourishing period of scientific production (e.g. Galileo, Toricelli and Malpighi), the Italian scientific production declined after the mid-seventeenth century in parallel with the stagnation of the Italian economy (Malanima, 2011). Thus while the ‘Republic of letters’ was possibly important during the early modern period, our results suggest that economic development is a key cause of scientific productivity.

### Ancient and medieval science

Finally, our study focused on early modern Europe. However, scientific production has been important in other societies as well, in particular in Ancient Greece and in the Arab and Persian worlds. Recent works in economic history suggests that these periods in these places were also characterised by increasing living standards (Morris, 2013; Ober, 2015; Özmucur & Pamuk, 2002). We thus wanted to test whether the association between affluence and scientific production holds for non-European societies, and whether increased prosperity could explain the rise of science during the ancient and medieval periods.

**Figure 6.**
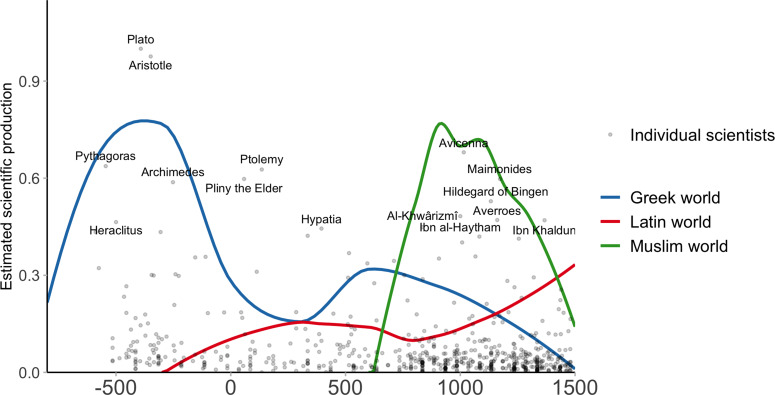
Estimated scientific production during the Antiquity and the Medieval period in the Western hemisphere.

During the medieval period (500–1500 CE), the most productive area was the Muslim world (see Figure 6). The Muslim area not only transmitted the Greek advances but also improved them in many ways and foreshadowed the development of the Western science (Chaney, 2018; Cohen, 2012; Huff, 2017; Rashed, 2002; Starr, 2013). Important examples of improvements are al-Khwarizmi's contribution to algebra and geometry, Ibn al-Haythan's theory of vision or Ibn Sahl's discovery of the law of refraction. Possibly the clearest evidence in favour of the advancement of science in the Muslim world is astronomy, in which there was a clear sense of progress, from the early criticisms of the Ptolemaic system by Ibn al-Haytham in the eleventh. c to the creation of new mathematical models by al-Bitruji (d. 1204), al-‘Urdi (d. 1266), al-Tusi (d. 1274), Qutb al-Din al-Shirazi (d. 1311) and Ibn al-Shatir (d. 1375) which were mathematically equivalent to the ones proposed by Copernicus a century and half later. This very high level of productivity is consistent with the higher level of urbanisation, GDP per capita and energy capture of the Muslim world during the period 700–1400 (Bosker et al., 2013; Morris, 2013; Pamuk & Shatzmiller, 2014).

Our results also shed light on the decline of Muslim science. In 1350, Muslim science was about to reach the level that Western scientists reached during the Scientific Revolution (in particular in astronomy with Al-Tusi). Yet not only did progress stop but also scientific production almost disappeared totally (Chaney, 2018; Cohen, 2012; Huff, 2017; Rashed, 2002; Starr, 2013). As historians of Muslim science Sabra writes: ‘It is precisely the high quality and sophisticated content of Islamic science that give poignancy to the problem of decline. The question is not why the efforts of Islamic scientists did not produce “the scientific revolution” (…) but why their work declined and eventually ceased to develop after the impressive flowering of the earlier centuries. Why, for instance, did algebra fail to make significant progress after the twelfth century? Why was the work of Ibn al-Haytham and Kamal al-Din in experimental optics not continued along lines drawn by these two mathematicians?’

Our results confirm these qualitative assessments (see Figure 6). Muslim countries reached a peak c. 1000 but started to decline very early on, c. 1150. Importantly, this is unlikely to be due to a measurement problem, as the most productive period is also the most ancient period.

In line with our main hypothesis, it must be noted that the decline of Muslim science parallels the economic decline of Muslim countries. From the eleventh century on, urbanisation and GDP per capita started to decline (Bosker et al., 2013; Morris, 2013; Pamuk & Shatzmiller, 2014), in particular because of environmental problems associated with a dry and difficult environment (Allen & Heldring, 2021; Montgomery, 2012; Ponting, 2007). In Iraq for instance, arguably the most affluent part of the Muslim world and the central hub of the scientific culture, GDP per capita decreased from 1400 in 720 to 900 in 1400 (Bosker et al., 2013; Morris, 2013; Pamuk & Shatzmiller, 2014; Van Bavel et al., 2018). This probably also contributed to the weakening of Islamic institutions. After the twelfth century, the average ruler duration started to decrease in the Muslim world (while it was increasing in Europe; Blaydes & Chaney, 2013).

In contrast to the Muslim societies, medieval Europe is often regarded as a backwater area, in particular in opposition to both Classical Greece and the Renaissance period. Our results are consistent with this negative view (see Figure 6). Up until the twelfth century, European scientific productivity is well below Muslim scientific productivity. These results are consistent with recent assessment of economic development during the early and central medieval periods (Bosker et al., 2013; Maddison, 2007; Milanovic, 2013; Morris, 2013). They are also consistent with the fact that while the most affluent parts of Europe (in particular northern Italy and Flanders) catch up with the Middle East around 1200, it is only around 1400 that Europe as a whole achieves a level of economic development similar to that of the Muslim world (Bosker et al., 2013; Maddison, 2007; Milanovic, 2013; Morris, 2013).

Our results show that scientific production started to rise as soon as the eleventh century, reached the level of the Muslim world around 1350 and quickly overtook it. This is in line with historians’ qualitative assessment (Cohen, 2012; Grant & Grant, 1996; Huff, 2017; Lindberg, 2010). For instance, historian of optics Mark Smith notes that the very fact that Alhacen's *De aspectibus* was translated in the thirteenth century suggests that European scientists had reached a level in optics high enough to be able to translate, read and understand this highly technical treatise:

First, there were translators available who were adequate to the task of rendering the text, with all its complexity, in a reasonably faithful manner. Second, there must have been a potential readership; otherwise, why undertake the laborious process of translating the text in the first place? We can thus assume that by no later than the beginning of the thirteenth century there was already a community of scholars in Europe prepared to assimilate or at least attempt to assimilate the De aspectibus. (Smith, 2014: 15)

Regarding total scientific production, and not per capita production, Europe overtook the Muslim world during the thirteenth century. This is because the population of Europe tripled during the central medieval period (1000–1300), and went from 22 to 66 million, while the population of the Muslim world stagnated around 20 million. So although European scientists were less productive individually up until 1350, Latin Europe was the most productive area from the thirteenth century on. For instance, although *De aspectibus* was produced by a Muslim scholar (Alhacen) in eleventh century Egypt, the book was much popular in Europe than in the Muslim world, with 18 complete copies in medieval Europe, against only one in the Muslim world. As Mark Smith notes: ‘It is possible, of course, that as-yet-undiscovered manuscripts of the Arabic text are squirreled away in badly cataloged collections, but it is highly unlikely that these will add significantly to the current total. As far as the *De aspectibus* is concerned, then, there was a significantly larger community of interest in medieval Europe than in the medieval Muslim world’ (Smith, 2014: 15).

## Discussion: the cultural evolution of science

We present the first estimates of scientific production during the late medieval and early modern period. They reveal important variations across countries and across periods. They also show a strong association between scientific productivity and economic development, as well as with artistic productivity, inclusive institutions and ascetic religiosity, suggesting a common underlying mindset of creativity (Baumard, 2019; McCloskey, 2016; Mokyr, 2016). Small but economically advanced societies such as England, Holland and Scotland, despite their limited population, were much more innovative than larger but less economically advanced societies, such as France, Italy or Russia (see Table 1). This result challenges a common idea in cultural evolution that population size is crucial in explaining creativity and innovation (Collard et al., 2013; Henrich, 2004; Kremer, 1993; Shennan, 2001).

It is worth noting that these results strikingly fit with the observations of contemporary observers. For instance, Voltaire, began his discussion of ‘great men’ with the three greatest – all of them English: Bacon, Locke and Newton (Mokyr, 2016: 68). Similarly, when Count Marsigli of Bologna visited the Royal Society in 1721, he was struck by the difference between Italy and England with regard to science: ‘[A]ll speculation unsupported by observation or experiment is utterly rejected. In England all study and teaching is based on fact’ (cited in Wootton, 2015).

In addition, our statistical modelling analysis suggests that economic development was a primary factor in the cultural evolution of science, whereas horizontal transmission does not seem to explain the rise of scientific production, as well as its geographic distribution. Beyond the specific case of science, this is an interesting result for the understanding of human cultural evolution. It converges with recent work in behavioural and political sciences, putting forward the explanatory power of economic development (Baumard et al., 2015; Inglehart, 2018; Martins & Baumard, 2020; Safra et al., 2019; Welzel, 2014) and demonstrates that ecological parameters (here and economic development) can drive the evolution of some aspects of human culture.

Several limitations need to be noted. First, our analysis is based on aggregate production at the country level. However, these aggregate estimates do not give a true estimate of the productivity of smaller units (but see Akaliyski et al., 2021). For instance, it is possible that the productivity of Ile-de-France (the region of Paris) is underestimated because it is pooled with the rest of France, which was much less developed. Similarly, the pooling of southern and northern Italy might downplay the burgeoning Italian Renaissance. Second, our indices of economic development are necessarily limited, and are likely to be revised in the future (Pleijt & Zanden, 2020). For instance, recent works suggest that the GDP per capita of Renaissance Italy has been overestimated. It is likely that northern Italy at its peak was not as rich as has been thought (Zanden et al., 2017), which fits with our own results showing a lower scientific productivity than during the English and Dutch golden ages. Third, GDP per capita is obviously a crude measure of human development, and a limited indicator of human capabilities. For instance, it is notable that Scandinavia was more productive scientifically than could be predicted from its GDP per capita. Other factors could play a role, such as individual freedom, education and gender equality, that are higher in north-western Europe (Santos Silva et al., 2017; Welzel, 2014).

Among the most discussed factors in the history of science, it is important to mention religion, and in particular Protestantism. Since the work of Max Weber and Robert K. Merton, Protestantism has often been cited as a possible cause of the Scientific Revolution. Protestant beliefs (in predestination), the work ethic, asceticism and the insistence on reading the Bible would have led Protestant countries to have a more favourable attitude to science (Cohen, 2012). The problem with such a theory is that Protestantism is not an exogenous factor. Even if there is an association between Protestantism and scientific production, this association could be the product of another hidden cause. In fact, religious asceticism, and in particular Protestantism, is also likely to be associated with scientific creativity because individuals with more long-term orientations are more likely to embrace a religion that values self-control, self-determination and investment in learning (Baumard et al., 2018; Baumard & Chevallier, 2015).

To test the association with Protestantism, we created two groups of countries based on the religious affiliation of the majority of the population after the Wars of Religion and the Treaty of Westphalia, when the borders of each religion were more and less settled. Protestant countries include England, Scotland, Germany, the Netherlands, Switzerland and Scandinavia. Catholic countries include France, Italy, Portugal, Spain and Belgium. In line with the life-history framework, our results show a strong association between per capita scientific production and Protestantism. In line with Weber and Merton's account, both Protestantism and GDP are significantly associated with scientific productivity in a bivariate model (*β* = 0.46 for Protestantism, *p* < 10^−3^, *β* = 0.30 for GDP per capita, *p* < 10^−3^). Again the correlational nature of these analyses does not allow us to make any causal claim.

More generally, studying the evolution of science over the very long term seems to tend to downplay the role of religion (Cohen, 2012; Grant & Grant, 1996; Needham, 1981; Stark, 2007). As shown by our results, Islam has been both associated with a very high scientific production (800–1200) as well as with a more limited scientific production (1200–1500). The same is true for Christianity, which is associated with a low scientific production during the medieval period, and a higher productivity during the early modern period. This results goes against standard hypotheses in history of science.

It is worth noting that our study does not demonstrate a causal link between economic development and scientific productivity. Changes in both prosperity and creative productivity could have been caused by another, hidden change in history. One candidate is institutional change: inclusive institutions could have caused a rise in both scientific productivity and economic development (Acemoglu & Robinson, 2012; North, 1990), generating a spurious correlation between scientific production and economic development. However, economic development could have generated both the rise of science and the rise of democracy. However, things could have gone the other way around, from economic development to inclusive institutions and scientific creativity. In fact, a growing body of work both in political science (Abramson & Boix, 2014; Boix & Stokes, 2003) and in the behavioural sciences (Martins & Baumard, 2020; Petersen & Aarøe, 2015; Safra et al., 2019) suggests that better life conditions are associated with higher level of interpersonal trust and higher support for inclusive institutions. In other words, institutional development, political freedom and inclusiveness are endogenous and accompany economic development (Boix & Stokes, 2003). At present, it is difficult to disentangle these hypotheses. In the remainder of the article, we discuss an important aspect of this question, namely that good theories of the Scientific Revolution must explain not only the link between economic development and scientific productivity, but also the link between scientific productivity and a new attitude towards novelty and exploration.

### Explaining the new attitudes towards innovation and exploration

Several interpretations of our results are possible. One possibility is simply that, in more economically advanced societies, individuals have more time, energy and resources to invest in scientific innovation. Science is indeed an expensive activity, because it requires a high level of human and material capital, but also because, until the end of the ninteenth century, it rarely brought substantial economic benefits. Our results suggest that rising living standards and economic prosperity further the advancement of science: scientific productivity was higher in the most affluent countries, and there is a positive association between living standards and both scientific and artistic productivity. One possible conclusion from these results could be that the Scientific Revolution is just the consequence of an increase of material resources.

Another interpretation is that, in addition to having more resources, north-western Europeans also had different preferences that were more conducive to innovation. The history of science, however, suggests that there was more than simply more time and more resources behind scientific advances. In his account of the Scientific Revolution, David Wootton (2015) observes that, in many cases, what prevented people from advancing their knowledge was not a lack of time or money, but the inability to question authorities:

Mondino de Liuzzi (1270–1326), for example, the author of the first medieval textbook on how to perform a dissection, had plenty of hands-on experience, but he still found at the base of the human brain the *rete mirabile* (miraculous network) of blood vessels that Galen claimed was there, despite the fact that it isn't there at all – it is only present in ungulates. (…) The first anatomist regularly to disagree with Galen on the basis of direct experience was Jacopo Berengario da Carpi, whose Anatomy was published in 1535, only a few years before Vesalius's Fabric. Only in a culture where the authority of the great classical authors such as Ptolemy and Galen had begun to be undermined could a project like Vesalius's Fabric be undertaken. (Wootton, 2015: 335)

Wootton makes a similar point about the ninteenth-century revolution in medicine. He points out that the germ theory of disease could have been discovered much earlier than the ninteenth century for the primary obstacle to progress, he argues, was not practical (Leeuwenhoek's microscopes worked well), nor theoretical (the germ theory of putrefaction was not difficult to formulate) but psychological and cultural (Wootton, 2015: 286).

More generally, as Wootton points out, before the sixteenth and seventeenth centuries, history was assumed to repeat itself, and tradition taken to provide a reliable guide to the future. The greatest achievements of civilisation were believed to lie not in the present or the future but in the past, in ancient Greece and classical Rome. In contrast, a new optimistic attitude towards the future and the possibility of progress gradually emerged in the sixteenth century.

This new attitude was summed up by Louis Le Roy (or Regius, 1510–1577) in 1575:

[T]here remayne more thinges to be sought out, then are alreadie invented, and founde. And let us not be so simple, as to attribute so much unto the Auncients, that wee beleeve that they have knowen all, and said all; without leaving anything to be said, by those that should come after them … Let us not thinke that nature hath given them all her good gifts, that she might be barren in time to come: … How many [secrets of nature] have bin first knowen and found out in this age? I say, new lands, new seas, new formes of men, maners, lawes, and customes; new diseases, and new remedies; new waies of the Heaven, and of the Ocean, never before found out; and new starres seen? yea, and how many remaine to be. (Cited in Wootton, 2015: 118)

A similar attitude can be found in writings by Bacon, Descartes and Galileo (Rossi, 2001; Wootton, 2015). This is reflected in the titles of the scientific books of the time: *Nova de universis philosophia* (Patrizi, 1591), *Novo theatro de machine* (Zonca, 1607), *Astronomia nova* (Kepler, 1609), *Discursi intorno a due nuove scienze* (Galileo, 1638), *De mundo nostro sublunari philosophia nova* (Gilbert, 1651). During the seventeenth century, more than 100 books used the term ‘novus’ in their title (Thorndike, 1971). As Mokyr (2016) notes, they no longer subscribed to ‘the Ecclesiastes view of history’, which holds that long-term change is impossible, because ‘there is nothing new under the sun’.

Seventeenth century intellectuals were also more open to creativity and innovation. As McCloskey notes, words such as ‘innovation’ and ‘novelty’ often used to have negative connotations before the seventeenth century. These same words started to be more positive, and emotional attachment to traditional ways of doing things progressively decreased (McCloskey, 2016: 94).

These examples suggest that the cultural change that occurred during the period conventionally identified as the Scientific Revolution consisted at least partially in changes in mindset or mentality, as if the obstacles to the spread of the new scientific ideas were psychological, rather than technical (e.g. the need for a new instrument) or economic (e.g. the need for more education, time, books, etc.). For instance, Galileo tells the story of a professor who refused to accept that the nerves were connected to the brain rather than the heart because this was at odds with Aristotle's explicit statement – and stood his ground even when he was shown the pathways of the nerves in a dissected cadaver (Wootton, 2015). He also reports that his close philosopher friend Cremonini refused to look through his telescope for the simple reason that Aristotle did not use telescopes, and they were therefore irrelevant (Wootton, 2015).

This shift in views on the progress of science predates actual scientific progress, which also argues for our hypothesis. In his famous study on the decline of magic, Keith Thomas noted that:

In many different spheres of life, the period saw the emergence of a new faith in the potentialities of human initiative. (…) The change was less a matter of positive technical progress than of an expectation of greater progress in the future. (…) It marked a break with the characteristic medieval attitude of contemplative resignation. (Thomas, 1971: 1184)

Thomas was struck that this optimism in the power of technical progress could not be based on actual evidence. ‘It is often said that witch-beliefs are a consequence of inadequate medical technique. But in England such beliefs declined before medical therapy had made much of an advance’ (Thomas, 1971: 1211). In the same way, the popularity of the work of Francis Bacon in the seventeenth century – that is, before the great wave of technical progress – attests that the English were very receptive towards optimistic ideas (Mokyr, 2016).

Similar observations can be made in the domain of technological innovation. As Howes (2016b) observes, eighteenth- and ninteenth-century innovators had an ‘improving mentality’, seeing room for improvement everywhere. Henry Dircks, an English engineer who improved steam engines and designed optical illusions, offered this very clear characterisation of this new mentality:

No work of art appears perfect to an enterprising mind. However simple its purpose, it may possibly be made lighter, stronger, more efficacious, or be done away with altogether. The man whose mind is thus constituted becomes an Inventor. (As cited in Howes, 2016a: 8)

Yet if north-western Europeans had different preferences that were more conducive to innovation, why this association with economic development and living standards? Recent work in behavioural sciences have found that resource abundance triggers cognitive exploration, curiosity, independent- and open-mindedness, whereas resource scarcity triggers conservatism and conformism, in order not to take the risk of an unfruitful exploration (Dubourg et al., 2021; Dubourg & Baumard, 2022; Jacquet et al., 2018; Nettle, 2018). This interpretation fits well with the fact that at the time of the Scientific Revolution, north-western European had a very specific psychology that was more future-oriented, with higher levels of social trust, lower levels of interpersonal violence, a greater interest in romantic love and a greater interest in parental investment (Baumard, 2018).

The rising living standards experienced by Europeans may thus have changed individuals’ psychology towards more optimism and non-conformism. As a result, Europeans would have started being more confident in their capacity to change the world, to propose new theories and to test them. In turn, this optimism may have made the experimental method and natural philosophy more appealing (despite the fact that, in the seventeenth century, there was as yet no evidence that experiments and science could really improve people's situation).

## Methods

### Scientific and artistic production

To collect the datasets, we used a top-down approach based on Wikipedia categories (Laouenan et al., 2022). First, we manually identified all the relevant Wikipedia categories (for instance, ‘eighteenth century Italian mathematicians’).

We only included individuals working in the ‘hard sciences’: mathematicians, physicists, astronomers, chemists, biologists, botanists, and zoologists (the first three categories account for more 90% of the sample). The rationale for this choice is that these domains correspond to the core domains of the seventeenth century Scientific Revolution. They also correspond to what economists call ‘useful knowledge’, the kind of knowledge involved in the manipulation of the material world, and the main source of growth during the Industrial Revolution (Jacob, 2014; Mokyr, 2016). In any case, the number of social scientists in Wikipedia before 1850 is too limited for a quantitative study. We also excluded physicians, because their biographies are mostly concerned not with scientific advances, but with their social role at the time. Moreover, given medical doctors’ poor record at increasing medical knowledge before the late ninteenth century, they arguably do not represent a good proxy for innovation (Wootton, 2007).

We also wanted to measure cultural creativity in general. We thus included all occupations that involve an important component of innovation and creativity: writers, philosophers, painters, musicians and sculptors. In contrast, we excluded rulers, military personnel, lawyers, religious leaders and physicians because these occupations arguably do not involve the same level of creativity. Some extra creative occupations could have been included, but were excluded owing to the small number of individuals in each category: engineers, geographers, explorers, cartographers or architects. In total, 22,943 individuals were included in the dataset (see Table S1).

We included in our sample all modern countries for which there were lists by nationality in science for the ninteenth century or before. Our final list includes the following countries: Belgium, UK, France, Germany, Italy, the Netherlands, Iberia (Spain and Portugal), Poland, Russia and Scandinavia (see Figure S1). We created two aggregated countries (Scandinavia and Iberia) because some environmental variables were only available at this level (see below, urbanisation).

Where country of citizenship (around 30% of the pages) was not specified on the individual's Wikidata item page, we inferred it using two methods. First, when possible, we referred to the Wikipedia category in which we had found the page (e.g. someone belonging to the category of ‘Italian mathematicians’ was considered Italian). When this was not possible, our code looked at the first words on the page and used the common structure ‘[Name] ([dates]) was a …’. A page beginning with ‘Galileo Galilei (15 February 1564 to 8 January 1642) was an Italian astronomer’ meant that Galileo was classified as Italian. Finally, we checked and completed by hand the most important dataset of this study, concerning Renaissance scientists.

We collected three proxies for the importance of an individual's contribution to the advancement of science (Gergaud et al., 2017):

1. Length: The number of bytes in the Wikipedia page (from ‘Page Information’) 2. Languages: The number of languages in which there was a page for the individual (from Wikidata) 3. Quotation: The number of Wikipedia pages containing a link toward this page (from the corresponding Wikidata item page).

To avoid a potential language bias leading to an overestimation of British production, we ran this study on the three largest Wikipedias: English, French and German. Contrary to our expectations, the datasets from the French and German Wikipedias were not always smaller than those from English Wikipedia (for instance, the German-language dataset of scientists is much larger than the English-language dataset, while the opposite is true for painters). Note also that previous work on notable individuals in Wikipedia has demonstrated that while a small portion of lesser-known individuals are present in non-English pages but absent in English pages, this is not the case for the most notable individuals (Gergaud et al., 2017).

As we record three proxies for each of the three languages, we have nine indicators of an individual's production (see Figure S3). None of these indices is perfect, and they all have their biases, so we combined them through a Principal Component Analysis (PCA), to compute the *Individual Scientific productivity*. The resulting values are neatly distributed as a power law with *α* = 1. This shape seems robust for different periods (e.g. scientists of antiquity) or different type of individuals (e.g. Renaissance artists).

Next, for a given year (here noted *y*), we computed the Total Scientific Product. Assuming an average peak productivity at 35, we used the following formula: National Scientific Product is the sum of the Individual Scientific Productivity of all individuals who reached the age of 35 between the year *y* − 25 and the year *y* + 25 in a given country.

To get per capita estimates, we divided the National Scientific Product by the estimate population in year *y* (estimates are taken from the *Atlas of World Population*; McEvedy et al., 1978). This provided demographic estimates for every century, or every 50 years. We thus performed a linear extrapolation to derive population figures at 10 year intervals for each country.

Because we were interested in the dynamics of the Scientific Revolution, which is usually dated around 1650, we chose 1300 as the starting date of our study and 1850 as the ending date.

### Environmental variables

In order to study the ecological determinants of scientific production, we used two measures of standard of living: GDP per capita (Broadberry et al., 2017) and urbanisation ratio (Bosker et al., 2013).

To test the importance of universities, we used the number of universities retrieved from Wikipedia's ‘List of medieval universities’ and ‘List of oldest universities in continuous operation’. As mentioned earlier, we also used the number of days of parliamentary sessions to test the effect of inclusive institutions, and the majority religion of the country, for the effect of Protestantism. We took these environmental variables as predictors, ran linear regressions of country-level cultural production against each variable, and computed correlation coefficients for production (taken as the mean of the *Z*-scores for the nine estimates).

## Data Availability

All the data and code are available in this repository: https://github.com/regicid/Code_scientific_revolution.

## Supplementary material

For supplementary material accompanying this paper visit https://doi.org/10.1017/ehs.2023.6. click here to view supplementary material
