# Supplementary material for: Quantifying the scientific revolution
Source: Evol Hum Sci. 2023 Apr 13;5:e19. doi: 10.1017/ehs.2023.6 (PMC10426016; doi:10.1017/ehs.2023.6)
Supplement: Supplementary file 1 [file S2513843X23000063sup001.pdf]

Supplementary Materials

1 Demography of the sample

We included all occupations that involve an important component of innovation and creativity: writers, philosophers, painters, musicians and sculptors. By contrast, we excluded rulers, military personnel, lawyers, religious leaders, and physicians because these occupations arguably do not involve the same level of creativity. Some extra creative occupations could have been included, but were excluded due to the small number of individuals in each category: engineers, geographers, explorers, cartographers, or architects. In total, 22,943 individuals were included in the dataset (see Table 1).

We included in our sample all modern countries for which there were lists by nationality in science for the 19th century or before. We created two aggregated countries (Scandinvia and Iberia) because some environmental variables were only available at this level (see below, urbanization).

As shown in Figure S1, national productivity differs markedly from per capita productivity (Figure 1, main manuscript).

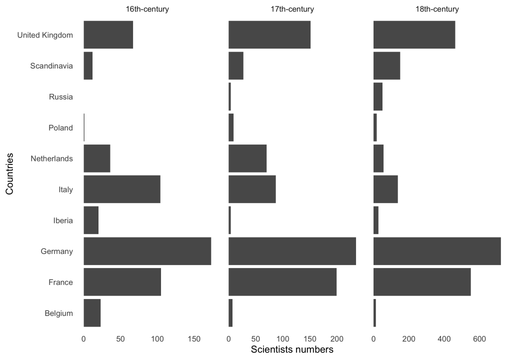

Figure 1: Number of individuals per country

Top 10 individuals

Names	Languages	Country	Birth	Death
Isaac Newton	207	United Kingdom	1643	1727
Galileo Galilei	176	Italy	1564	1642
Charles Darwin	175	United Kingdom	1809	1882
Nicolaus Copernicus	150	Germany	1473	1543
Leonhard Euler	142	Germany	1707	1783
Dmitri Mendeleev	135	Russia	1834	1907
Carl Friedrich Gauss	134	Germany	1777	1855
Alfred Nobel	133	Scandinavia	1833	1896
Johannes Kepler	132	Germany	1571	1630
Gottfried Wilhelm Leibniz	132	Germany	1646	1716

Random sample of individuals with a median score

Names	Languages	Country	Birth	Death
Marc-Auguste Pictet	9	Germany	1752	1825
Wilhelm Keferstein	9	Germany	1833	1870
Paolo Frisi	9	Italy	1728	1784
Heinrich Schumacher	9	Germany	1757	1830
Jacques Rohault	9	France	1618	1672
Gabrio Piola	9	Italy	1794	1850
Carl Osten-Sacken	9	Russia	1828	1906
Friedrich Weber	9	Germany	1781	1823
Samuel Klingenstierna	9	Scandinavia	1698	1765
Nicolas Charles Seringe	9	France	1776	1858

Random sample of individuals with a low score

Names	Languages	Country	Birth	Death
Richard Pendlebury	1	United Kingdom	1847	1902
Thomas Rudd	1	United Kingdom	1583	1656
Walter Trevelyan	1	United Kingdom	1797	1879
James Douglas Dickson	1	United Kingdom	1849	1931
Alexander Yersin	1	Switzerland	1825	1863
Carl Goldschmidt	1	Germany	1807	1851
Antonio Filippo Ciucci	1	Italy	1650	1710
Adam Anderson	1	United Kingdom	1783	1846
William Campion	1	United Kingdom	1820	1896
Gustav Schwartz	1	Austria	1809	1890

Table 1: Scientists ranked by the importance of their contributions to the advancement of science

Discipline	Number of pages
scientists	3017
composers	2311
painters	9366
writers	6631
philosophers	441
sculptors	1177

Table 2: Number of individuals per occupation

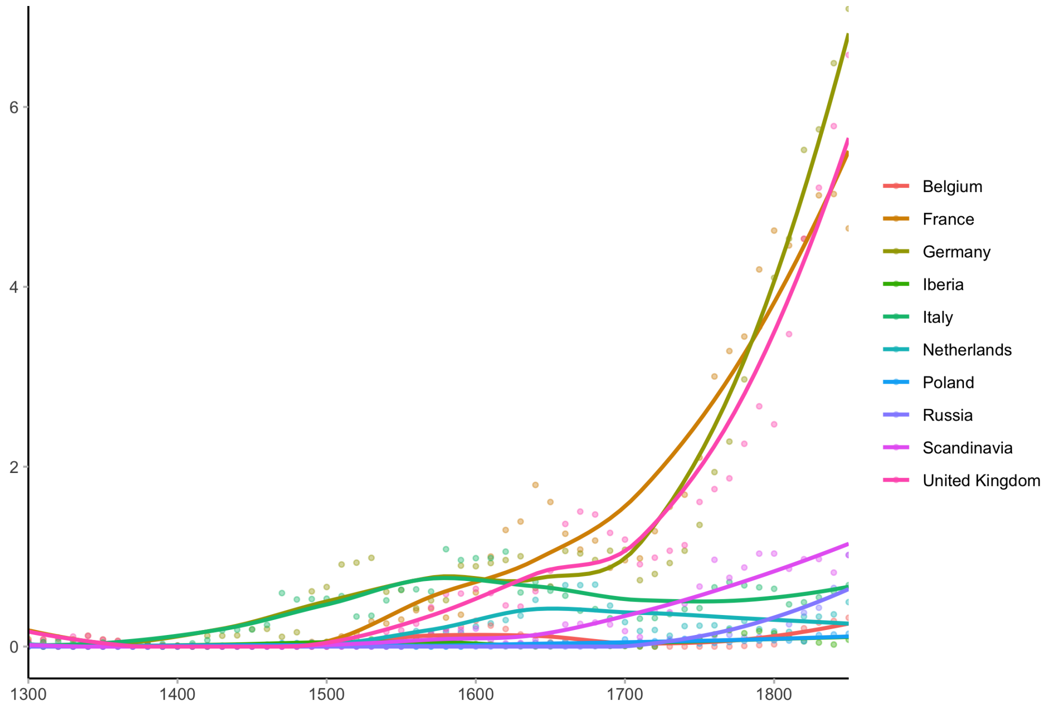

Figure 2: National scientific production (1500 – 1850)

2 Per capita estimates

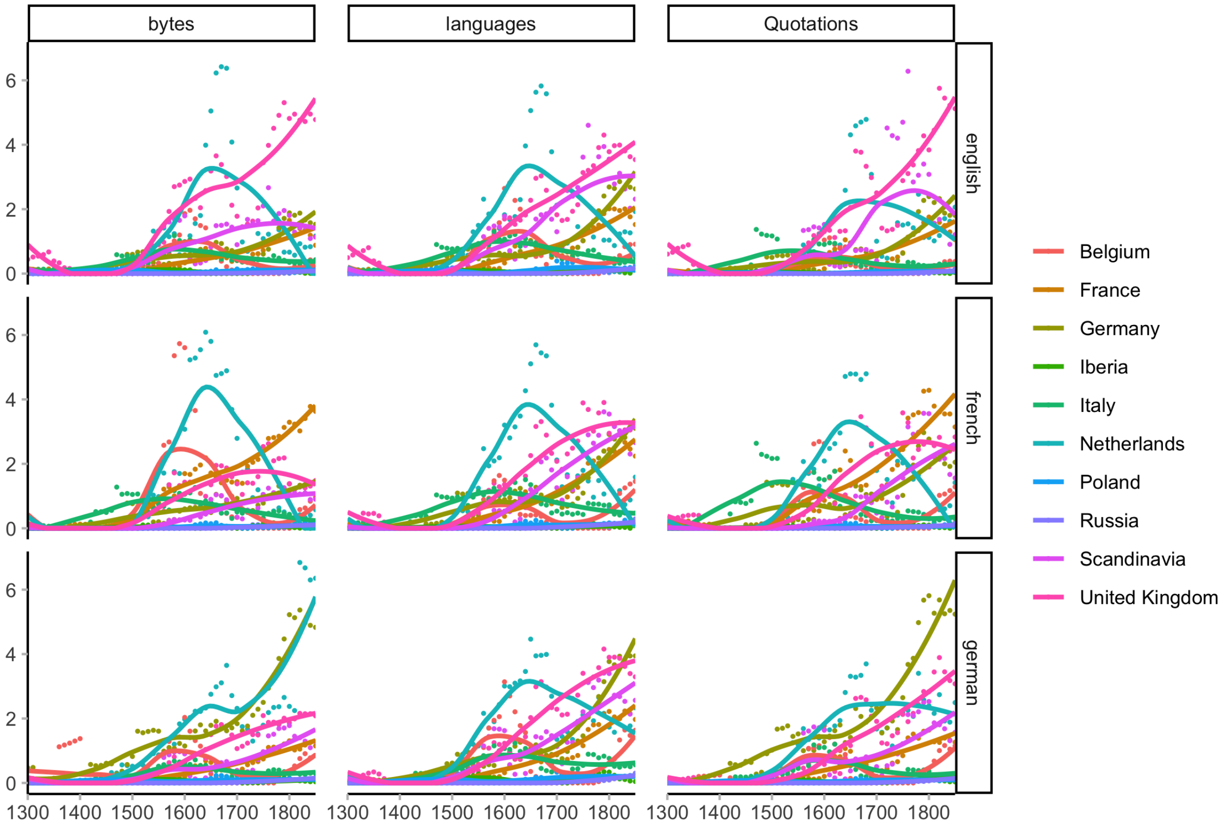

Figure 3: Estimated scientific production per capita per language and per proxy (1500-1850)

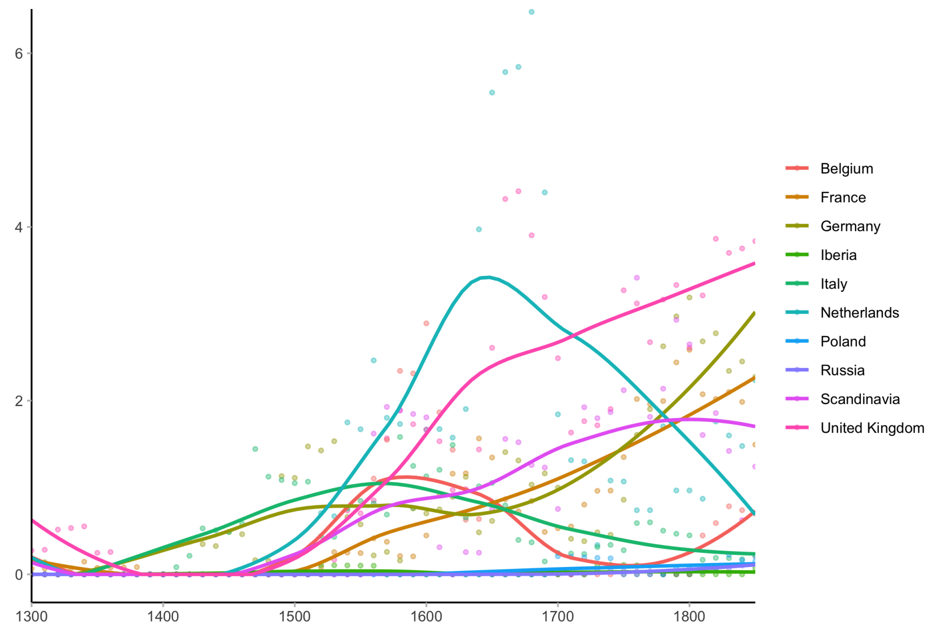

Figure 4: Estimated scientific Production per capita (top 10%)

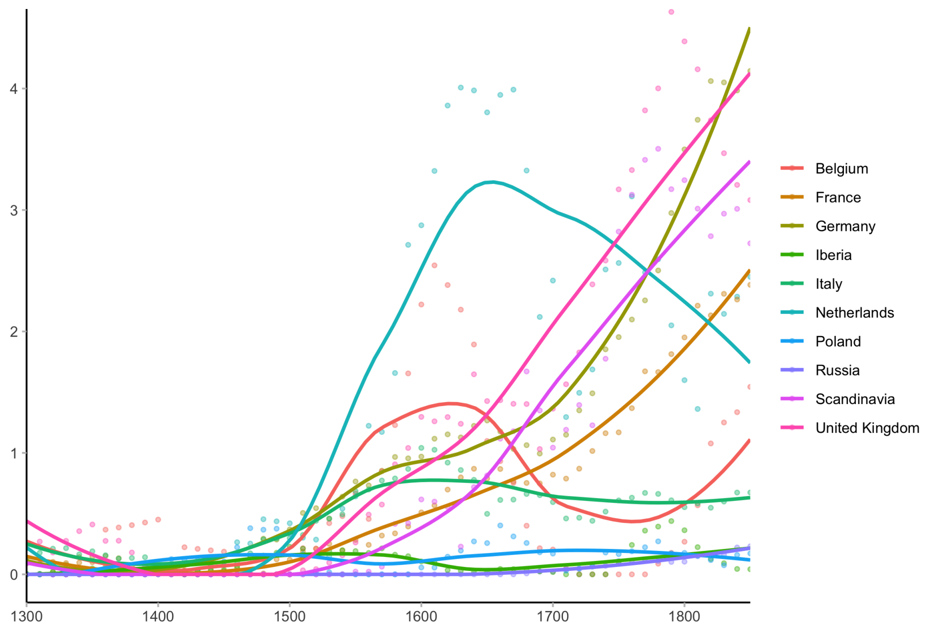

Figure 5: Estimated cientific Production per capita (bottom 90%)

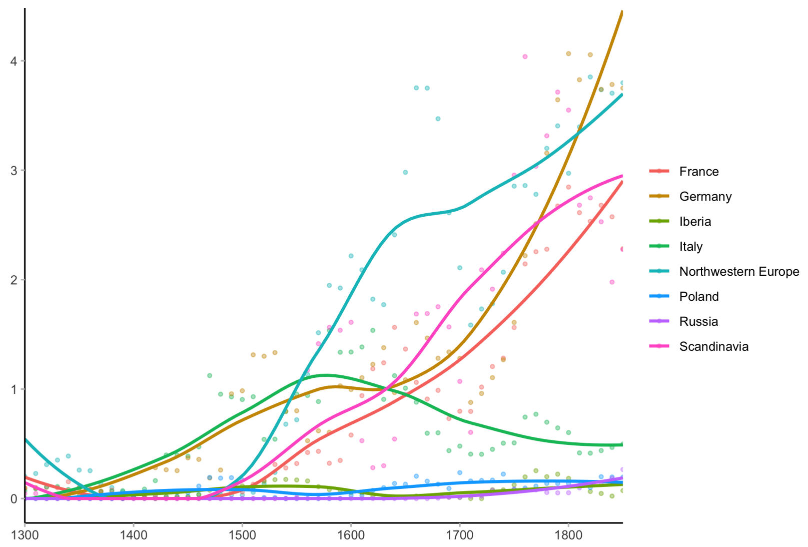

Figure 6: Estimated cientific Production per capita including Northwestern Europe (i.e., England, Scotland, Wales, Ireland, the Netherlands and Belgium combined)

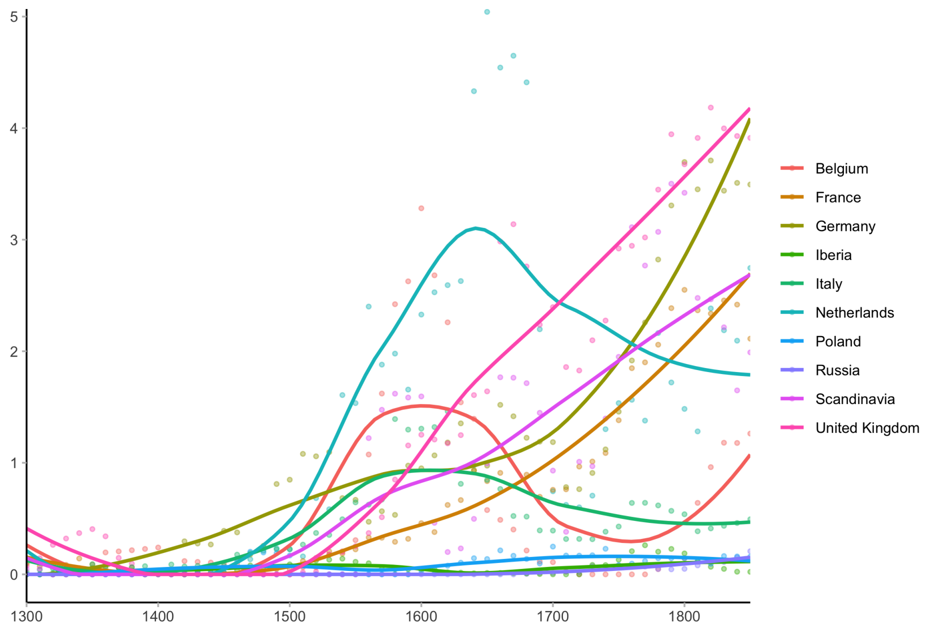

Figure 7: Estimated cientific Production per capita ('scientist' first occupation only)

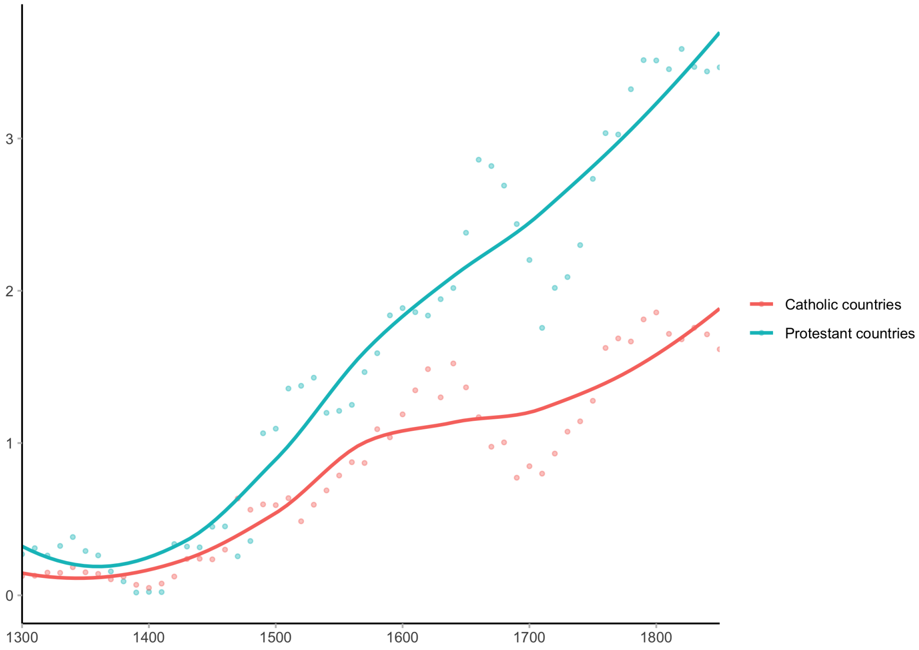

Figure 8: Estimated scientific production per capita for Catholic and Protestant countries

3 Statistical modelling

We chose to model the data by *Partially observed Markov process*, implemented by the R package *Pomp* (1). This framework is simulation-based: to search for the maximum-likelihood parameters, Pomp only requires to specify a way to *simulate* the data (with a given set of parameters), instead of a way to *evaluate* the likelihood of these parameters (which is computed by Monte-Carlo methods, by simulating a lot of trajectories and measuring how it fits with data). This gives a considerable flexibility, allowing to specify much more explicit models, though at a large computational cost.

Each model is fitted by *iterated filtering*. One way to understand the algorithm is draw an analogy with natural selection. Starting by an initial guess (a vector of parameter values), the algorithm creates a "population" of parameters vectors by perturbating the values, thus creating variation. Each vector can be thought as a genotype. A particle filter (or sequential Monte-Carlo) then estimates the likelihood of each parameter vector, a measure of the compatibility between the parameters and the data, which gives an analogue of fitness. Each vector then reproduces in proportion to their likelihood, forming a

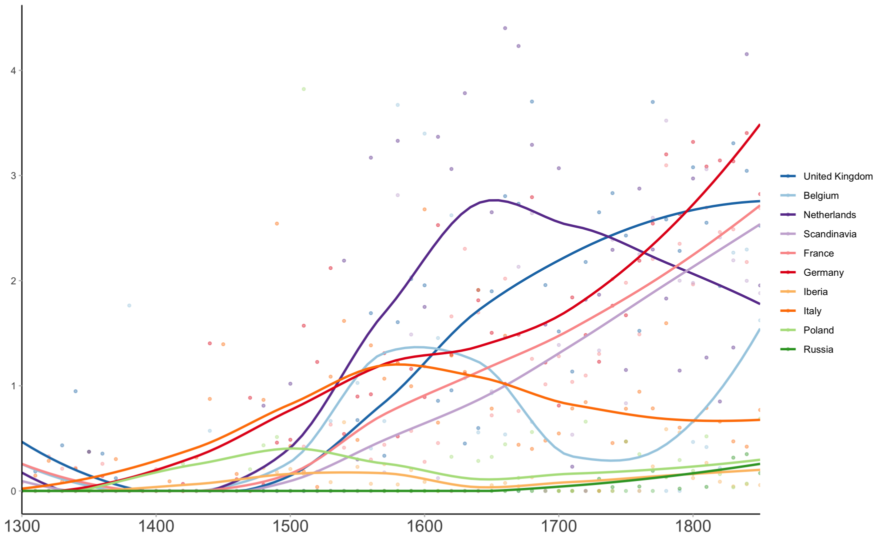

Figure 9: Scientific production per capita estimated without the English Wikipedia

new population. This process is iterated, the perturbations being progressively cooled down, as we are interested in a model without artificial perturbations. Just as natural selection typically brings the genotype around a fitness peak, the process is predicted to stabilize on (possibly local) maximum likelihood parameters. To be sure to find the global maximum likelihood parameters, this process is repeated with different starting points, all across the range of reasonable guesses. We also chose to log-transform the data, as it provides a more intelligible analysis (without changing the results qualitatively).

The model with the lowest AIC is the (C) one, which only takes into account autoregression and the effect of GDP ($AIC = 123.2$ for (C), $AIC > 124.9$ for all other models). Using BIC gives the same result (with ($BIC = 142.6$ for (C), $BIC > 147$ for all other models). The likelihood ratio test allows us to reject the (B) model for the (A) one ($p < 10^-6$), which confirms that the data are autocorrelated. The (C) model also explains the data significantly better than (B) ($p = .008$), which confirms the role of GDP. Aside, no model without the impact of GDP is able to reject the (B) model: (D), (F) and (J) are not significantly better than (B) ($p = .504$, $p = .301$ and $p = .266$, respectively), which casts doubt on the role of cumulated production, horizontal diffusion and horizontal cumulated diffusion to account for scientific production. Similarly, no complexification of the (C) model is able to reject it: neither by adding an interaction between GDP and cumulated production ((E) vs (C): $p = .865$), nor by adding horizontal diffusion ((G) vs (C): $p = .91$) or an interaction between GDP and horizontal diffusion ((I) vs (C): $p = 634$). The most complex model

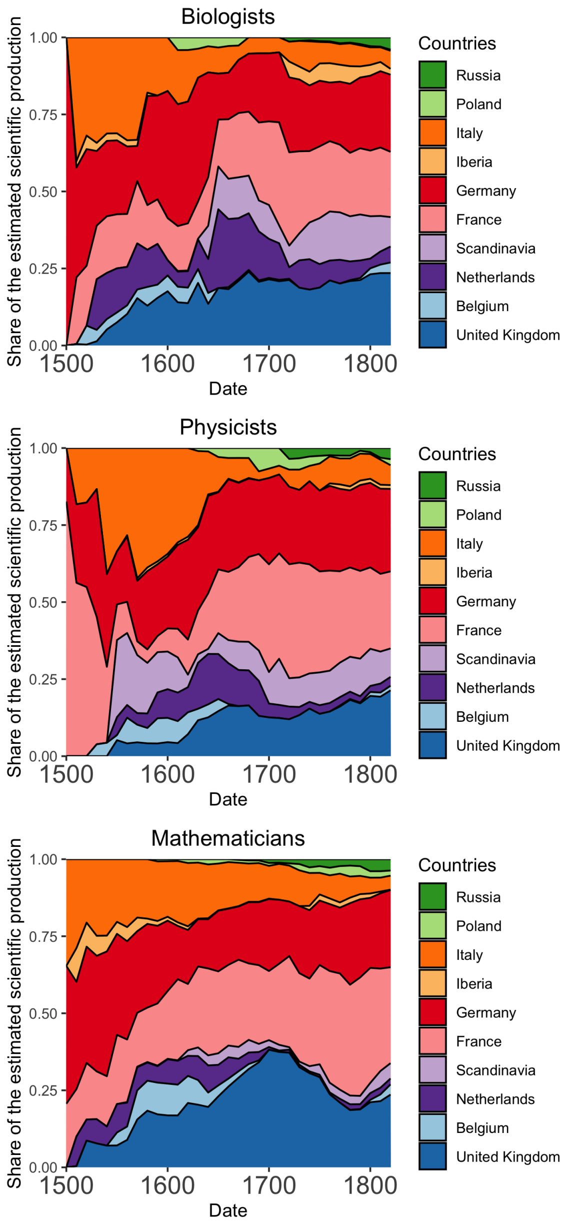

Figure 10: Evolution of the share of estimated scientific production per country and per discipline (1300 - 1850)

(K) has a higher likelihood than C, which is normal: adding parameters to a model can only increase the likelihood. Yet, it is also insufficient to reject the model C ($p = .687$).

	AIC	BIC	Log-likelihood
A	304.79	321.42	-146.40
B	148.87	168.26	-67.43
C	136.94	159.10	-60.47
D	149.98	172.14	-66.99
E	138.88	163.81	-60.44
F	148.73	170.89	-66.36
G	138.55	163.49	-60.28
H	148.39	170.56	-66.20
I	138.48	163.42	-60.24
J	145.92	165.32	-65.96
K	138.76	172.01	-57.38

Table 3: Scores of the models: Akaike information criterion, Bayesian information criterion and log-likelihood

3.1 Likelihood ratio tests

A Pomp model requires two components:

i) The "state process", a stochastic relation between scientific production at time t and $t+1$, allowing to simulate trajectories.

   GDP and N_j (the production of other countries) are treated as covariables, time-varying given environment parameters. $\epsilon \sim \mathcal{N}(1, \sigma)$ produces a multiplicative noise prone to produce the fat-tailed data we observe. $\epsilon \sim \mathcal{N}(1, \sigma_2)$ is a classic white noise.

ii) A "measurement model": the data (n_{obs}) are assumed to be a stochastic function of a latent unobserved variable n, which we are interested in. In our case, we simply assumed that we observe the reality up to a white noise : $n_{obs} \sim \mathcal{N}(n, \sigma_{obs})$.

We considered the following models :

A) The null model assumes that scientific production, during a time step t and for a region i, is independent of past production, of production from other regions, or of GDP. A region always has a mean production c, with a variance driven from the random variable $\epsilon' \sim \mathcal{N}(0, \sigma')$ corresponding to white noise.

$$n_i^t = c + \epsilon'$$

B) The model with vertical transmission assumes that scientific production, during a time step t and for a region i, depends on n_i^{t-1}, the scientific production at time $t-1$.

$$n_i^t = c + z \cdot n_i^{t-1} \cdot \epsilon + \epsilon'$$

C) The model with GDP assumes that scientific production, during a time step t and for a region i, depends on GDP_i^t the GDP of that region at time t.

$$n_i^t = c + z \cdot n_i^{t-1} \cdot \epsilon + \alpha \cdot GDP_i^t + \epsilon'$$

D) The model with cumulated vertical transmission assumes that scientific production, during a time step t and for a region i, depends on the scientific production of the region at times $j < t$.

$$n_i^t = c + z \cdot n_i^{t-1} \cdot \epsilon + \sum_{j<t} n_i^j \cdot e + \epsilon'$$

E) The model with cumulated vertical transmission tied to GDP assumes that scientific production, during a time step t and for a region i, depends on GDP_i^t the GDP of that region at time t, and on the interaction between this GDP and the scientific production of the region at times $j < t$.

$$n_i^t = c + z \cdot n_i^{t-1} \cdot \epsilon + \alpha \cdot GDP_i^t + \sum_{j<t} n_i^j \cdot f \cdot GDP_i^t + \epsilon'$$

F) The model with horizontal transmission assumes that scientific production, during a time step t and for a region i, depends on N_j^{t-1} the scientific production of other regions j at time $t-1$, and $D_{i,j}$ the distance between regions i and j.

$$n_i^t = c + z \cdot n_i^{t-1} \cdot \epsilon + \sum_{j \neq i} \frac{n_j^{t-1}}{D_{i,j}^2} d + \epsilon'$$

G) The same model, assuming that scientific production also depends on GDP:

$$n_i^t = c + z \cdot n_i^{t-1} \cdot \epsilon + \alpha \cdot GDP_i^t + \sum_{j \neq i} \frac{n_j^{t-1}}{D_{i,j}^2} d + \epsilon'$$

H) The model with horizontal transmission tied to GDP assumes that scientific production, during a time step t and for a region i, depends on GDP_i^t the GDP of that region at time t, N_j^{t-1} the scientific production of other regions j at time $t-1$, and $D_{i,j}$ the distance between regions i and j.

$$n_i^t = c + z \cdot n_i^{t-1} \cdot \epsilon + \sum_{j \neq i} \frac{n_j^{t-1}}{D_{i,j}^2} (b \cdot GDP_i^t) + \epsilon'$$

I) The same model, assuming that scientific production also depends on GDP:

$$n_i^t = c + z \cdot n_i^{t-1} \cdot \epsilon + \alpha \cdot GDP_i^t + \sum_{j \neq i} \frac{n_j^{t-1}}{D_{i,j}^2} (b \cdot GDP_i^t) + \epsilon'$$

J) The model with cumulated horizontal transmission assumes that scientific production, during a time step t and for a region i, depends on n_j^k the scientific productions of other regions j at time k, and $D_{i,j}$ the distance between regions i and j.

$$n_i^t = c + z \cdot n_i^{t-1} \cdot \epsilon + \sum_{j \neq i} \sum_{k<t} \frac{n_j^k}{D_{i,j}^2} d + \epsilon'$$

K) The most complex model, all other models being included in this one, is then :

$$n_i^t = z \cdot n_i^{t-1} \cdot \epsilon + \alpha \cdot GDP_i^t + \sum_{j \neq i} \frac{N_j^{t-1}}{D_{i,j}^2} (d + b \cdot GDP_i^t) + N_i^{t-1} (e + f \cdot GDP_i^t) + c + \epsilon'$$

In 3.1, we present the p-values resulting of all possible likelihood ratio tests. These tests are only doable on nested models, hence a scarce matrix.

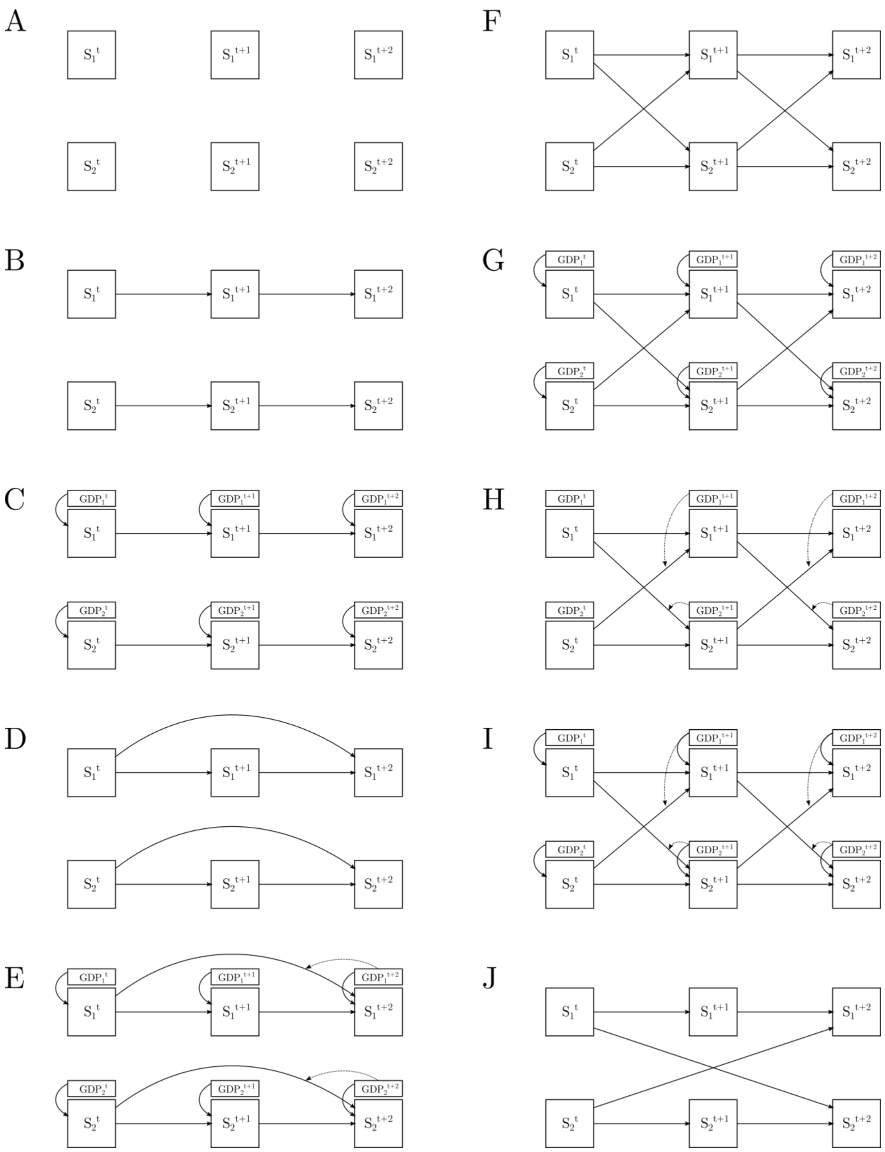

Figure 11: Models of scientific production and diffusion. Each box A to J represents one of the models proposed to explain scientific production. Each square represents the scientific production of a region over the course of 50 years. Only two regions (named region 1 and region 2) are represented here, and only three time steps (named t, t+1 and t+2) are represented here. The solid arrows represent the direct influence of either the scientific production of a region on the scientific production of another region, or the level of economic development of a region (represented here by the GDP) on its scientific production. The dotted arrows represent the influence of GDP on the importance of scientific diffusion between regions (horizontal) or time step (vertical). Model K, which combines all the hypotheses, is not shown.

	B	C	D	E	F	G	H	I	J	K
A	$< 10^{-6}$ ***	$< 10^{-6}$ ***	$< 10^{-6}$ ***	$< 10^{-6}$ ***	$< 10^{-6}$ ***	$< 10^{-6}$ ***	$< 10^{-6}$ ***	$< 10^{-6}$ ***	$< 10^{-6}$ ***	$< 10^{-6}$ ***
B		0.008 **	0.504	0.03 *	0.301	0.028 *	0.266	0.027 *	0.225	0.122
C				0.865		0.662		0.634		0.687
D										0.087 .
E										0.548
F						0.014 *				0.11
G										0.575
H								0.015 *		0.117
I										0.582
J										0.127

Table 4: Results of all possible likelihood ratio tests, with null hypothesis in rows and alternative hypothesis in columns

3.2 Convergence analysis

To verify that the algorithm is indeed reaching a unique maximum likelihood point, whatever the starting point, we can plot the evolution of each parameter value through iterations (see Fig. 12). Here, note that the variance parameters (σ, σ_2 and σ_{obs}) are squared, hence the symmetry across zero).

However, there is still some variability among estimates. To investigate why, we can plot pairwise plots of the different estimates (Fig. 13, which give more insights on the likelihood landscape. Here, we can observe two reasons for this variability. First, pure noise due to the algorithm, moving the estimate slightly away from the maximum likelihood, just like in evolutionary biology, mutational load moves phenotypes slighly away from the optimum. We can indeed see in the first row of Fig. 13 that log-likelihood plotted against each parameter forms a bell curve. Second, we can observe for instance that the higher the parameter a is estimated, the lower z and c are. This indicates "ridges" in the likelihood landscape, which are prone to generate variability in estimates.

References

[1] King, A. A., Nguyen, D. & Ionides, E. L., 2015 Statistical inference for partially observed markov processes via the r package pomp. *arXiv preprint arXiv:1509.00503* .

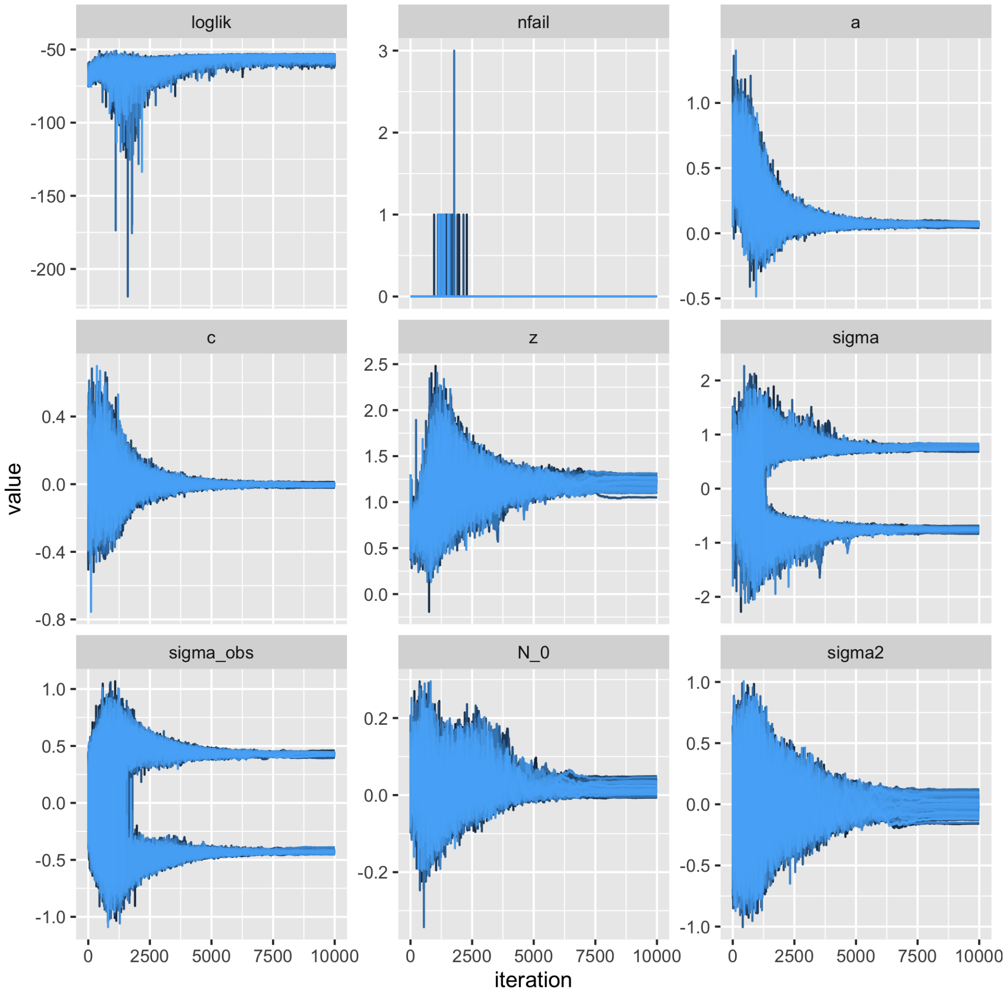

Figure 12: Convergence diagnostic for the model C (200 different starting points, $Np = 20000, Nmif = 10000$)

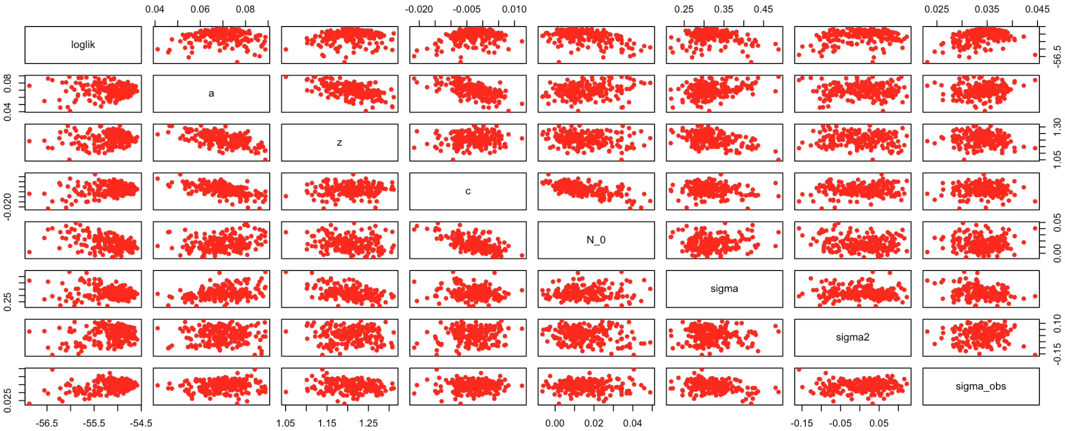

Figure 13: Pairwise estimates of all runs of the (C) model
